# Reclamation of Astragalus By-Product through Dietary Inclusion in Ruminant Diets: Effects on Growth Performance, Nutrient Digestibility, Rumen Fermentation, Blood Biochemical Parameters, and Humoral Immune Response in Sheep

**DOI:** 10.1155/2019/8530961

**Published:** 2019-09-03

**Authors:** Abedin Abdallah, Pei Zhang, Abdul-Halim Abubakari, Evera Elemba, Qingzhen Zhong, Zewei Sun

**Affiliations:** ^1^Key Laboratory of Animal Nutrition and Feed Science, Key Laboratory of Animal Production, Product Quality and Security, College of Animal Science and Technology, Jilin Agricultural University, Changchun, China; ^2^Faculty of Agriculture, University for Development Studies, Tamale, Ghana; ^3^College of Food Science and Engineering, Jilin Agricultural University, Changchun, Jilin 130118, China

## Abstract

This study was conducted to investigate the effects of Astragalus by-product (ABP) through dietary supplementation at different levels on performance, nutrient digestibility, rumen fermentation, blood metabolites, and immune response in sheep. Twenty-four Doper × Small Tail Han ewes (6-7 months of age; 29.07 ± 2.28 kg initial body weight) were randomly assigned to one of three treatments for a 47 d feeding period. Treatments consisted of the sheep diet supplemented with 0% ABP-control, 10% ABP, or 15% ABP of the diet (dry matter basis). Blood samples were collected on days 0, 15, 30, and 45 of the feeding period. APB supplementation did not affect growth performance and apparent digestibility of organic matter, crude protein, and acid detergent fibre (*P* > 0.05). However, ether extract digestibility was decreased in the 10% ABP group and increased in the 15% ABP group (*P* < 0.001), and both 10% ABP and 15% ABP decreased the neutral detergent fibre digestibility (*P*=0.005). Feeding ABP increased rumen pH (*P* < 0.001) and ammonia N (*P* < 0.001) and decreased concentrations of acetate (*P*=0.007) and propionate (*P*=0.001) which resultantly increased the acetate-to-propionate ratio (*P* < 0.001) in ruminal fluid. There were no interaction effects between treatment and sampling time for plasma metabolites and immunity (*P* > 0.05). However, inclusion of dietary 10% ABP decreased concentrations of plasma cholesterol (*P*=0.043). Also, plasma concentrations of low-density lipoprotein decreased on days 30 and 45 (*P*=0.017) of the feeding period. Metabolite concentrations of total protein, albumin, globulin, blood urea N, glucose, triglyceride, and high-density lipoprotein cholesterol and humoral immune indicators were not affected (*P* > 0.05) by dietary ABP supplementation. The results suggest that ABP could be reclaimed through dietary inclusion in animal feed since it had beneficial effects on rumen fermentation patterns and lipid metabolism and had no adverse effects on performance and humoral immunity in sheep.

## 1. Introduction

*Astragalus membranaceus*, also known as Huangqi in Chinese and Radix Astragali in Latin, is a widely used immunomodulating herb mainly in traditional Chinese medicine. The root of *A. membranaceus* contains over 100 bioactive compounds prominent among which include polysaccharides, flavonoids, amino acids, and saponins [1, 2]. Several studies have indicated the immunomodulatory, cardioprotective, antiviral, antioxidative, hepatoprotective, antitumor, antidiabetic, and anti-inflammatory properties of *A. membranaceus* mainly due to the activities of the bioactive compounds contained in them [1, 3, 4].

In China, the annual yield of herbal by-products including those of *A. membranaceus* is approximately 30 million tons and mostly disposed of through combustion, heaping in the open or sanitary burial, causing serious environmental pollution, especially in water quality [5–7]. For example, most methods used to extract Astragalus polysaccharides involve water extraction and alcohol precipitation. These methods not only leave many other bioactive constituents behind, but also produce a large amount of Astragalus by-products (ABP) [4, 8]. Several reports show that ABP still contains many nutrients such as crude protein, amino acids, crude fibre, crude fat, and lignin and also includes abundant medicinal components such as terpenoids, alkaloids, and saponins [4]. For instance, a polysaccharide extraction yield of 12.93% with a purity of 93.27% was realized when polysaccharide was extracted from ABP with water [9].

Several studies have attempted to reclaim herbal by-products as feed additives, preparation of activated carbon, papermaking, cultivation of edible fungi, preparation of ethanol, or fermentation for the purpose of limiting infections such as diarrhea [5, 10]. In an experiment to evaluate the therapeutic effects of the fermentation supernatant of herbal residue (composed of parts of *Pseudostellaria heterophylla*, *Dioscorea opposita*, *Hordeum vulgare*, *Crataegus pinnatifida*, *Citrus reticulata*, and Jianweixiaoshi sts), the fermentation supernatant scavenged 77.8% of 2,2-diphenyl-1-picrylhydrazyl (DPPH), 78% of O_2_•, 36.7% of •OH, 39% of Fe^2+^ chelation, and 716 mg/L reducing power. In the same experiment, the fermentation supernatant inhibited diarrhea at a high rate (56%, *p* < 0.05), significantly boosted the destruction of antibiotic-based bacterial diversity, and reestablished the prevalence of *Lactobacillus johnsonii* in the treatment of antibiotic-associated diarrhea in mice [10]. In another study, ABP improved immunity and promoted the digestion ability of white ducks resulting in a corresponding increase in body weight and weight of the ducks' immune organs [3].

Sheep is one of the most important sources of meat produced and consumed worldwide. About 70% of the world's population consumes sheep and goat meat as part of their regular diet and, in several countries, are the main products of traditional dishes [11]. So far, no attempt has been made to reuse ABP as dietary feed additives in sheep production. Therefore, in the present study, ABP was incorporated into sheep diet to evaluate its effect on growth performance, apparent nutrient digestibility, rumen fermentation, blood biochemical parameters, and immune response in Doper × Small Tail Han hybrid sheep and to exploit this dietary option as a potential means of ABP reclamation.

## 2. Materials and Methods

All experimental protocols involving animals were approved by the Institutional Committee for Animal use and Ethics of the College of Animal Nutrition and Feed Science of Jilin Agricultural University and also in agreement with the provincial rules and regulations.

### 2.1. Astragalus By-Product

The fresh by-product of *A. membranaceus* root used in this study was obtained from Xiuzheng Pharmaceutical Company Limited in Tonghua City, China. The by-product was air-dried and then pulverized to pass a 2 mm screen.

### 2.2. Experimental Design, Animals, Diets, and Housing

A total of 24 6- to 7-month-old crossbred Doper × Small Tailed Han ewes with an average initial body weight of 29.07 ± 2.28 kg were allocated to 3 dietary treatments with 8 repetitions (8 animals in each treatment) in a completely randomized design by stratified randomization based on their live body weights. Thus, animals were weighed at the beginning of the experiment and randomly assigned to treatments consisting of diets supplemented with various levels of ABP as follows: diets with 0% ABP-control, 10% ABP, and 15% ABP (Table 1). The animals were arranged in a covered area and individually allotted on a suspended slatted floor into individual pens with free access to food and drinking water in a feedlot system. The experiment lasted for 47 days and was preceded by a 30-day adaptation period (a total of 77 days), during which time ewes were weighed, identified, and treated for ecto- and endoparasites, and diet adaptation was performed.

The experimental diets were formulated to be isonitrogenous and isoenergetic and on the basis of the National Research Council [12] recommendations for an average daily weight gain of 200 g/day (Table 1). The diets were fed twice daily at 08:00 and 16:00 h and were offered as roughage first and then concentrate. The leftovers were weighed daily, and the amount of supplied feed was adjusted to allow for leftovers of 10 to 20% refusal. ABP and dietary samples were analyzed for dry matter (DM), crude protein (CP), and ether extract (EE) according to the method of the Association of Official Analytical Chemist [13], and acid detergent fibre (ADF) and neutral detergent fibre (NDF) according to the method described by Van Soest et al. [14]. At the end of the feeding period, sheep were weighed after 3 h fasting and then transported to a commercial slaughterhouse for slaughtering according to acceptable standards.

### 2.3. Sampling and Measurements

The sheep were weighed before the morning feeding on the first and last days of the feeding period to calculate the average daily gain (ADG), and the daily feed supply and orts for each sheep were recorded to calculate the average daily feed intake (ADFI). The feed conversion ratio (FCR) was expressed as feed consumption per unit of body weight gain.

Nine days of apparent nutrient digestibility measurements was conducted from days 37 to 45 at the end of the feeding period, and 6 d sampling (total feces) was preceded by 3 d adaptation. Feed refusals and total fecal samples were collected before the morning feeding from each sheep and weighed. Then, 10 mL hydrochloric acid (2.877 mol/L) was added to 10% of the fresh feces from each sheep to prevent ammonia N volatilization. At the end of the digestibility trial period, the feed refusal and feces samples were composited, dried at 65°C for 72 h, weighed, and milled through a 1 mm sieve for chemical analysis. The samples were analyzed according to the Association of Official Analytical Chemists standard procedures [15] for crude protein (CP, Kjeldahl procedure; method 976.06), ether extract (EE, method 920.29), and crude ash (550°C in a furnace for 6 h; method 942.05). Acid detergent fibre (ADF) and neutral detergent fibre (NDF) were determined by the method of Van Soest et al. [14] inclusive of residual ash, according to the filter bag technique with addition of amylase and sodium sulfite.

Ruminal fluid samples were collected from the rumen immediately after sheep were slaughtered. Ruminal pH was measured immediately using a portable type pH meter (Testo 205, Testo AG, Lenzkirch, Germany). Afterwards, the ruminal fluid was filtered through 4 layers of cheesecloth and the extracts were centrifuged at 10,000 ×g for 15 min at 4°C. The supernatant of the ruminal fluid (15 ml) was transferred into tubes containing 0.3 ml of 500 mg/g sulfuric acid, mixed, and stored at −20°C for NH_3_-N analysis by the phenol-hypochlorite method, as described by Broderick and Kang [16]. The remaining strained ruminal fluid samples, 10 ml, were mixed with 1 ml of 250 mg/g metaphosphoric acid and stored at −20°C for later determination of volatile fatty acid profiles by gas chromatography [17].

Before the morning feeding on days 0, 15, 30, and 45 of the feeding period, blood samples were collected from the jugular vein of each sheep into 5 ml heparinized collection tubes and centrifuged at 3000 × g for 15 min at 4°C. The supernatant (plasma) was collected and frozen at −20°C pending further analysis. For blood biochemical parameters, total protein (TP), albumin (ALB), globulin (GLB), blood urea N (BUN), glucose (GLU), triglyceride (TG), total cholesterol (CHOL), high-density lipoprotein cholesterol (HDL-CH), and low-density lipoprotein cholesterol (LDL-CH) concentrations were determined using an automatic biochemistry analyzer (SYNCHRON CX5 PRO, Beckman Coulter, Fullerton, CA, USA). For immune response, immunoglobulins (IgG, IgA, and IgM) were determined using an ELISA test kit (Diagnostic Automation/Cortez Diagnostics, Inc., Los Angeles, CA, USA).

### 2.4. Statistical Analysis

The data of growth performance of sheep, apparent nutrient digestibility, and rumen fermentation parameters were analyzed using a general linear model, followed by Duncan's multiple range tests [18]. The data for plasma parameters of sheep were analyzed using the mixed model procedure [19] with a model consisting of treatment, sampling time, and treatment × time interaction as fixed effects and animal as the random effect. Measurements obtained from each sheep at different sampling times were treated as repeated measures. Polynomial analysis was conducted to determine the linear or quadratic response to the increasing ABP dosage in the diet. The means of each trait were compared by Turkey multiple comparisons and presented with the standard error of the mean. Differences were considered statistically significant if *P* ≤ 0.05.

## 3. Results

### 3.1. Growth Performance and Nutrient Digestibility

Table 2 shows the effects of increasing levels of ABP on sheep growth performance attributes. Dietary supplementation of ABP marginally improved the ADG and ADFI of sheep even though the differences were not significant (*P*=0.771;  0.253). Conversely, FCR among dietary treatments did not differ significantly (*P*=0.911). Feeding ABP decreased the apparent digestibility of dietary NDF in a linear (*P*=0.008) and quadratic (*P*=0.034) manner. Moreover, dietary ABP supplementation linearly (*P*=0.013) and quadratically (*P* < 0.001) increased the apparent digestibility of dietary EE (*P* < 0.001) in comparison with the control group. Dietary CP, CF, ADF, and ash were not affected by ABP supplementation (*P* > 0.05).

### 3.2. Rumen Fermentation Parameters

Rumen fermentation parameters were affected by the treatment diet (Table 3). The pH of the rumen liquid varied between 5.8 and 6.6 and had a linear (*P* < 0.001) and quadratic (*P*=0.055) response to dietary ABP supplementation. The pH was greater (*P* < 0.001) for sheep in the 10% ABP and 15% ABP groups than for sheep in the control group. The molar proportions of acetate and propionate were significantly decreased (*P*=0.007; *P*=0.001) by ABP. The butyrate concentration varied between 5.6 mmol/L and 9 mmol/L and had a linear (*P*=0.023) response to dietary ABP addition. The highest acetate-to-propionate ratio was observed in the 10% ABP dietary group (3.64) and that of the 15% ABP group was significantly higher than for the control group (*P* < 0.001). Feeding ABP markedly increased the ammonia N concentration of the rumen linearly (*P* < 0.001) and quadratically (*P*=0.003).

### 3.3. Blood Biochemical Parameters

There were no interaction effects between treatment and sampling time for plasma metabolites and humoral immunity (*P* > 0.05) among dietary treatments (Table 4). However, inclusion of dietary ABP significantly decreased (*P*=0.043) concentrations of plasma cholesterol of sheep in the 10% ABP dietary group. Also, there was a time effect on LDL-CH as plasma concentrations decreased with time and significantly so on days 30 and 45 (*P*=0.017) of the feeding period. Metabolite concentrations of TP, ALB, GLB, BUN, GLU, TG, and HDL-CH and humoral immune indicators IgG, IgM, and IgA were not affected (*P* > 0.05) by dietary supplementation on ABP.

## 4. Discussion

This study is among the first to investigate the effects of the by-products of Astragalus, a traditional Chinese herbal medicine as a feed supplement in the diet on growth performance, apparent nutrient digestibility, rumen fermentation, blood biochemical parameters, and humoral immune response in sheep. The primary aim of this study was to provide evidence that it is more feasible to use ABP as a feed additive in animal production rather than piling and burning, which results in environmental pollution. This is also in sync with previous attempts that have been made to increase rumen development and weight gain of postweaned lambs by nutritional strategies instead of performance-enhancing drugs considering the high demand for organic animal products in the consumer markets [1]. In the present study, we found that dietary supplementation of ABP did not affect the growth performance indicators in sheep, even though there was a marginal improvement in ADG and ADFI. Similar to the present observations, Zhong et al. [1] had found that dietary supplementation of *A. membranaceus* root did not affect dry matter intake, ADG, and FCR in lambs. Furthermore, a study on the effects of dietary *A. membranaceus* root powder addition at various levels on the growth performance of broiler chicks showed that dietary ADFI was not affected [20]. This is an indication that dietary inclusion of ABP does not affect the palatability of feed. In contrast to our results, previous studies with nonruminants showed that dietary *A. membranaceus* supplementation increased dry matter intake and ADG in pigs [21] and improved the ADG and FCR in broilers [20]. The observed differences may have been due to the different microenvironments in the digestive tract between ruminants and nonruminants, as well as the different dose levels of Astragalus.

Several studies have shown that phytogenic-based feed additives could increase villi length and decrease crypt depth in the jejunum and colon [22, 23]. Apparent digestibility of protein may be increased by improving the digestive capability of the prececum since the dominant proportion of total fecal protein is bacterial proteins. An improved prececal digestive capability decreases the flux of fermentable material into the hindgut and consequently decreasing postileal microbial growth and fecal bacterial growth [24]. Also, most medicinal plants have been reported to contain essential oils that may improve nutrients digestibility in animals [25]. However, we found that dietary ABP supplementation did not affect the apparent digestibility of CP, ADF, and ash in the present experiment. This result is consistent with that of Zhong et al. [1] who reported that dietary inclusion of 50 g/kg *Astragalus membranaceus* root had no effect on apparent nutrient digestibility in growing lambs. Furthermore, Qiao et al. [26] reported that extracts of Fructus Ligustri Lucidi, a traditional herbal medicine supplied at 100, 300, and 500 mg/kg dry matter, had no effect on CP, ADF, and NDF digestibility in sheep. In contrast, El-Ashry et al. [25] found that dietary addition of three different medicinal plants in the diet improved nutrient digestibility at 8 months of the feeding trial. This variation may have been due to the different total feeding time periods in the two experiments. Dietary EE and NDF affect digestion, metabolism, and microbial activity in animals [27]. In the present study, the inclusion of dietary ABP resulted in a decreased apparent NDF digestibility in the 10% ABP (from 69.40% to 54.78%) and 15% ABP (from 69.40% to 56.31%) dietary groups and increased apparent ether extract digestibility in the 15% ABP (from 55.56% to 72.32%) dietary group. Similar results were observed by Ghasemi et al. [27] who reported that replacement of lucerne hay with pistachio by-product either partially or fully decreased the apparent NDF and increased ether extract digestibility in sheep. It is worth noting that decreased dietary NDF to 22.9% of DM did not affect cellulolytic bacteria in the rumen of lactating dairy cows [28]. In the present study, the levels of NDF in the treatment groups were higher than this amount. The decreased NDF digestibility may have been due to the increase in fibre content as the content of ABP increased. These results suggest that dietary ABP inclusion does not have adverse effects on nutrient digestibility in sheep.

One of the most important indicators of the microbial ecosystem of the rumen is the ruminal pH. A more acidic microbial ecosystem poses a threat to the establishment of a balanced microbial population and also decreases microbial attachment which affects the digestion of fibre [1, 29, 30]. In the present experiment, dietary inclusion of ABP significantly increased the pH of ruminal fluid from 5.83 in the control group to 6.51 in the 15% ABP group. This pH falls within the optimal pH range stipulated for the rumen environment and rumen fermentation [31] in sheep. This indicates that dietary ABP inclusion has the potential to regulate and stabilize the microbial ecosystem in the rumen.

Ammonia nitrogen, NH_3_-N, is an essential indicator for the synthesis of microbial protein in the rumen since a bulk of the rumen bacteria use NH_3_ as a source of N [32, 33]. The minimum concentration of NH_3_-N required for maintaining an optimum microbial activity in the rumen is 5 mg/dl. As such, a higher concentration results from a more rapid ruminal degradation of proteins and/or reduced transport through the rumen wall [33]. In the current study, increasing dietary ABP addition correspondingly increased NH_3_-N in the rumen of sheep, suggesting that protein degradation increased with increasing ABP supplementation. However, this trend was not observed in the current experiment as CP digestibility was unaffected. In contrast, ruminal NH_3_-N concentration was unaffected when dietary Astragalus polysaccharide and Astragalus root were added to lambs' diet [1]. Also, Soltan et al. [34] observed that dietary inclusion of 25 and 50 g/kg DM of *Moringa oleifera* root bark, a herbal medicine, in growing lambs' diet did not affect the NH_3_-N concentration in the rumen. The increased NH_3_-N levels in the present study are probably as a result of lower contents of saponins (major active compounds in Astragalus) in the by-product as saponins have been suggested to decrease ammonia levels in the rumen [35].

In this study, dietary ABP inclusion significantly decreased the acetate and propionate concentrations and significantly increased the acetate-to-propionate ratio in the rumen while butyrate concentration remained unaffected even though a marginal decline was recorded. Several studies using herbal medicines as feed additives in animal production showed changes in VFA formation. For example, the molar proportion of acetate in ruminal fluid increased and that of butyrate decreased while those of total VFA and propionate as well as acetate : propionate proportion were unaffected when 500 g/kg *Urtica cannabina* was mixed with 500 g/kg concentrate and fed to growing lambs [36], indicating inconsistency with the current study. In another study, ruminal molar concentrations of total VFA, acetate, and acetate : propionate proportion increased while proportions of propionate, butyrate, isobutyrate, valerate, and isovalerate were unaffected when *Moringa oleifera* root bark was fed to lambs [34]. However, slightly consistent with the present experiment is that of Zhong et al. [1] who found that dietary inclusion of Astragalus root did not affect ruminal propionate and butyrate proportions although they had a decreasing tendency. Moreover, Ghasemi et al. [27] observed lower molar concentrations of ruminal acetate and propionate + isobutyrate, and no effect on butyrate when high and low levels of pistachio by-products were added to sheep diets, as was observed in the present experiment. The lower VFA concentrations in the present study were probably due to lower microbial activity in the rumen [27].

Concentrations of TP, ALB, BUN, and GLU are generally suggested to be determinants of protein synthesis and metabolism which are relevant for growth performance [35, 37]. Also, blood GLU concentration is generally regarded as the energy supply indicator in animals [38]. Kim et al. [39] suggested that increased TP and ALB concentrations indicate that more proteins are synthesized and absorbed, and decreased BUN indicate that more proteins are synthesized with amino acids since BUN is a product of protein degradation. In the present study, TP, ALB, BUN, and GLU concentrations were not affected by dietary ABP addition at the end of the feeding trial, indicating that ABP did not affect the synthesis and metabolism of protein. This is also reflected in the growth performance indicators in the present experiment as growth performance was not affected by dietary ABP addition. This is consistent with the findings of Zhong et al. [1] who reported that dietary inclusion of Astragalus root in lambs' diet did not affect TP, ALB, and BUN concentrations in the blood. Concentrations of blood metabolites such as TG, CHOL, HDL-CH, and LDL-CH are related to lipid N metabolism [1, 40]. Higher plasma concentrations of CHOL and TG are considered risk factors related to coronary heart diseases and atherosclerosis in humans [37, 41]. Moreover, the key function of plasma lipoproteins is the transportation of lipids from secreting organs like the intestine and liver to peripheral tissues [42, 43]. HDL-CH transports lipids from tissues to the liver and thus known as “good cholesterol,” whereas LDL-CH is largely related to the low plasma cholesteryl esters transfer activity and thus known as “bad cholesterol” [42]. For example, Cornier et al. [44] found that elevated levels of cholesterol, especially of LDL-CH, increased the risk of cardiovascular diseases. Several traditional Chinese herbal medicines have been suggested to affect lipid metabolism and consequently inhibit blood lipid-related diseases. For example, dietary Astragalus root extract supplementation decreased serum and hepatic TG and CHOL concentrations in rat liver [45]. Also, Astragalus and *Lycium barbarum* polysaccharides have been reported to lower total CHOL, LDL-CH, and very LDL-CH in rats [46, 47]. In accordance with the previous studies, dietary ABP supplementation decreased the plasma CHOL concentrations of sheep in the present experiment. Concentrations of TG and LDL-CH were also decreased in the 10% ABP group even though the differences were not significant from the control group. This indicates that dietary ABP could effectively metabolize lipids and thus prevent lipid-related diseases.

An important indicator of humoral immunity in animals is the level of plasma antibodies which mainly include immunoglobulins G (IgG), M (IgM), and A (IgA). They defend the extravascular compartment against pathogenic viruses and microorganisms [37, 48]. Several flavonoid-containing herbal medicines including *A. membranaceus* have been reported to enhance immune function [1, 24]. Furthermore, previous research has indicated that Astragalus polysaccharides could enhance humoral immunity in chicks [49–51]. In the present study, dietary ABP inclusion did not affect the humoral immune response in sheep. A possible reason could be that ABP levels in the present study were suboptimal. Similar findings were reported by Che et al. [52] who found that humoral immunity indicators IgG, IgM, and IgA were not affected when 2.5, 5.0, and 7.5% pulverized *A. membranaceus* stem and leaf fibre were fed to weaned pigs. This result suggests that ABP supplementation has no adverse effect on the immune integrity of sheep.

## 5. Conclusions

The findings of the current experiment suggest that dietary inclusion of *Astragalus membranaceus* by-products in diet had beneficial effects on rumen fermentation patterns and lipid metabolism and had the potential to improve growth performance in sheep. Additionally, no adverse effects on the production performance and immunity traits in sheep were recorded according to the results of the present study. It can be concluded that it is more useful to use ABP as a novel natural dietary feed additive in animal production systems similar to those of the present study, as opposed to disposal by piling and/or burning which causes serious environmental pollution.

## Figures and Tables

**Table 1:** Ingredients and chemical composition of the experimental diets.

Item	Dietary treatments^1^		
	Control	10% ABP	15% ABP
*Ingredient (g/kg of DM)*			
*Leymus chinensis*	258	250	220
Corn straw	175	145	145
Astragalus by-product	0	100	150
Molasses	20	20	20
Corn	260	259	278
Distillers dried grains with solubles	140	69	40
Corn gluten meal	99	115	97
Premix^2^	5	4	7
Urea	8	4	8
Sodium chloride	8	8	8
Calcium carbonate	15	15	15
Dicalcium phosphate	4	4	4
Sodium bicarbonate	8	8	8

*Chemical composition (g/kg DM)* ^*3*^			
Dry matter (g/kg fresh weight)	887	887	887
Crude protein	124	124	124
Neutral detergent fibre	437	436	430
Acid detergent fibre	260	269	273
Metabolizable energy (Mj/kg)	10	10	10
Calcium	8	8	8
Phosphorus	5	4	4

^1^ABP, Astragalus by-product; ^2^supplied per kg of diets: vitamin A, 15,000 IU; vitamin D, 2,000 IU; vitamin E, 55 IU; Fe, 50 mg; Co, 0.2 mg; Cu, 12.0 mg; Se, 0.5 mg; Mn, 50 mg; I, 0.55 mg; Zn, 25 mg; ^3^nutrient levels were all measured values except metabolizable energy.

**Table 2:** Growth performance and apparent nutrient digestibility of sheep fed diets supplemented with different levels of dietary Astragalus by-product.

Items^1^	Dietary treatments^2^			SEM	*P* value		
	Control	10% ABP	15% ABP		Diet	Linear	Quadratic
*Growth performance*							
ADG (kg/d)	0.12	0.13	0.13	0.005	0.771	0.620	0.637
ADFI (kg/d)	1.48	1.56	1.57	0.024	0.253	0.162	0.423
FCR	12.41	11.95	12.47	0.500	0.911	0.969	0.674

*Apparent nutrient digestibility (%)*							
OM	49.62	37.73	40.92	2.392	0.086	0.124	0.111
CP	70.58	65.70	67.44	0.988	0.099	0.179	0.096
EE	55.56^b^	24.50^c^	72.32^a^	5.831	<0.001	0.013	<0.001
ADF	45.42	30.90	39.32	2.970	0.110	0.377	0.062
NDF	69.40^a^	54.78^b^	56.31^b^	2.370	0.005	0.008	0.034

^a,b,c^Means within a row with different subscripts differ when *P* < 0.05; ^1^ADG, average daily gain; ADFI, average daily feed intake; FCR, feed conversion ratio; OM, organic matter; CP, crude protein; EE, ether extract; ADF, acid detergent fibre; NDF, neutral detergent fibre; ^2^ABP, Astragalus by-product; SEM, standard error of the mean.

**Table 3:** PH value and volatile fatty acid (VFA) concentration in ruminal fluid of sheep fed diets supplemented with different levels of dietary Astragalus by-product.

Items	Dietary treatments^1^			SEM	*P* value		
	Control	10% ABP	15% ABP		Diet	Linear	Quadratic
Rumen pH	5.83^b^	6.47^a^	6.51^a^	0.114	<0.001	<0.001	0.055
Acetic acid (mmol/L)	59.29^a^	40.72^b^	36.7^b^	3.877	0.007	0.003	0.134
Propionic acid (mmol/L)	22.58^a^	11.2^b^	11.25^b^	1.999	0.001	0.001	0.011
Butyric acid (mmol/L)	8.91	6.81	5.67	0.606	0.059	0.023	0.627
Acetic/propionic acid	2.63^c^	3.64^a^	3.27^b^	0.151	<0.001	0.001	<0.001
NH_3_-N (mg/dL)	3.51^c^	4.38^b^	7.13^a^	0.329	<0.001	<0.001	0.003

^a,b,c^Means within a row with different subscripts differ when *P* < 0.05; ^1^ABP, Astragalus by-product; SEM, standard error of the mean.

**Table 4:** Blood biochemical parameters and humoral immune response of sheep fed diets supplemented with different levels of dietary Astragalus by-product.

Items^1^	Dietary treatments^2^			SEM	Time^3^				*P* value				
	Control	10% ABP	15% ABP		D0	D15	D30	D45	DT	T	DT × T	*L*	*Q*
*Blood biochemical parameters*													
TP (g/L)	66.86	59.63	64.58	0.491	65.49	61.34	63.72	64.21	0.071	0.535	0.621	0.726	0.428
ALB (g/L)	35.58	32.81	35.51	0.669	35.09	33.09	34.67	35.68	0.184	0.437	0.449	0.437	0.245
GLB (g/L)	31.29	26.82	29.08	0.734	30.41	28.25	29.05	28.54	0.111	0.620	0.862	0.703	0.785
BUN (mmol/L)	6.17	5.42	6.48	0.231	5.99	6.22	6.76	5.11	0.125	0.077	0.206	0.145	0.627
GLU (mmol/L)	4.07	3.76	4.09	0.080	4.18	3.71	4.00	4.02	0.255	0.225	0.507	0.318	0.514
TG (mmol/L)	0.39	0.33	0.41	0.017	0.37	0.36	0.45	0.33	0.357	0.052	0.734	0.953	0.579
CHOL (mmol/L)	1.86^a^	1.41^b^	1.76^ab^	0.059	1.93	1.51	1.57	1.69	0.043	0.052	0.709	0.704	0.611
HDL-CH (mmol/L)	1.20	1.07	1.33	0.040	1.24	1.19	1.22	1.15	0.156	0.642	0.405	0.217	0.166
LDL-CH (mmol/L)	0.57	0.44	0.45	0.022	0.58^a^	0.45^ab^	0.43^b^	0.49^b^	0.285	0.017	0.375	0.143	0.286

*Immune response*													
IgG (*μ*g/mL)	126.72	127.25	122.69	5.287	138.95	115.70	113.25	134.32	0.904	0.390	0.960	0.772	0.765
IgM (*μ*g/mL)	364.69	363.40	361.72	9.664	405.67	347.71	342.74	356.97	0.988	0.301	0.649	0.103	0.278
IgA (*μ*g/mL)	54.54	52.06	54.41	1.812	56.33	54.60	51.52	52.23	0.682	0.760	0.941	0.992	0.574

^a,b^Means within a row with different subscripts differ when *P* < 0.05; ^1^TP, total protein; ALB, albumin; GLB, globulin; BUN, blood urea nitrogen; GLU, glucose; TG, triglyceride; CHOL, cholesterol; HDL-CH, high-density lipoprotein cholesterol; LDL-CH, low-density lipoprotein cholesterol; IgG, immunoglobulin G; IgM, immunoglobulin M; IgA, immunoglobulin A; ^2^ABP, Astragalus by-product; ^3^D, day; DT, dietary treatment; T, time; SEM, standard error of the mean.

## Data Availability

No data were used to support this study.

## References

[1] Zhong R. Z., Yu M., Liu H. W., Sun H. X., Cao Y., Zhou D. W. (2012). Effects of dietary *Astragalus* polysaccharide and *Astragalus membranaceus* root supplementation on growth performance, rumen fermentation, immune responses, and antioxidant status of lambs. *Animal Feed Science and Technology*.

[2] Zhang J., Xie X., Li C., Fu P. (2009). Systematic review of the renal protective effect of *Astragalus membranaceus* (root) on diabetic nephropathy in animal models. *Journal of Ethnopharmacology*.

[3] Zhu Z. Y., Li Y., Sun H. Q. (2016). Screening of Cordyceps strains and optimization of its solid-state fermentation conditions on bioconversion of Astragalus residue. *Cellulose Chemistry and Technology*.

[4] Sun H.-Q., Zhu Z.-Y., Yang X.-Y., Meng M., Dai L.-C., Zhang Y.-M. (2017). Preliminary characterization and immunostimulatory activity of a novel functional polysaccharide from Astragalus residue fermented by *Paecilomyces sinensis*. *RSC Advances*.

[5] Meng F., Yang S., Wang X. (2017). Reclamation of Chinese herb residues using probiotics and evaluation of their beneficial effect on pathogen infection. *Journal of Infection and Public Health*.

[6] Feng N., Zhang Y., Fan W., Zhu M. (2018). Enhancement of methylbenzene adsorption capacity through cetyl trimethyl ammonium bromide-modified activated carbon derived from Astragalus residue. *IOP Conference Series: Earth and Environmental Science*.

[7] Abedin A., Pei Z., Qingzhen Z., Zewei S. (2019). Application of traditional Chinese herbal medicine by-products as dietary feed supplements and antibiotic replacements in animal production. *Current Drug Metabolism*.

[8] Liu Y., Liu W., Li J. (2019). A polysaccharide extracted from *Astragalus membranaceus* residue improves cognitive dysfunction by altering gut microbiota in diabetic mice. *Carbohydrate Polymers*.

[9] Guo T., Wang Y., Zhu Y. L., Zhang Z. (2013). The reutilization of herbal residues. *Advanced Materials Research*.

[10] Meng F., Chen T., Ma D. (2017). Reclamation of herb residues using probiotics and their therapeutic effect on diarrhea. *Mediators of Inflammation*.

[11] Teixeira A., Fernandes A., Pereira E., Manuel A., Rodrigues S. (2017). Effect of salting and ripening on the physicochemical and sensory quality of goat and sheep cured legs. *Meat Science*.

[12] NRC (2010). *Guide for the Care and Use of Laboratory Animals*.

[13] AOAC (2002). *Official Methods of Analysis*.

[14] Van Soest P. J., Robertson J. B., Lewis B. A. (1991). Methods for dietary fiber, neutral detergent fiber, and nonstarch polysaccharides in relation to animal nutrition. *Journal of Dairy Science*.

[15] AOAC (1990). *Official Methods of Analysis of the Association of Official Analytical Chemists*.

[16] Broderick G. A., Kang J. H. (1980). Automated simultaneous determination of ammonia and total amino acids in ruminal fluid and in vitro media. *Journal of Dairy Science*.

[17] Isac M. D., García M. A., Aguilera J. F., Alcaide E. M. (1994). A comparative study of nutrient digestibility, kinetics of digestion and passage and rumen fermentation pattern in goats and sheep offered medium quality forages at the maintenance level of feeding. *Archiv für Tierernaehrung*.

[18] SAS (2002). *SAS/STAT® User’s Guide: Statistics*.

[19] Littell R. C., Milliken G. A., Stroup W. W., Wolfinger R. D. (1996). *SAS System for Mixed Models Wiley Online Library*.

[20] Wang H. F., Yang W. R., Yang H. W. (2010). Effects ofAstragalus membranaceuson growth performance, carcass characteristics, and antioxidant status of broiler chickens. *Acta Agriculturae Scandinavica, Section A—Animal Science*.

[21] Hu Y., Xu C., Wang Y., Li Y., Liu J., Feng J. (2006). Effect of dried roots of *Astragalus membranaceus* in the diets of young growing pigs on growth performance and immune function. *Journal of Animal and Feed Sciences*.

[22] Namkung H., Gong M. L. J., Yu H., Cottrill M., de Lange C. F. M. (2004). Impact of feeding blends of organic acids and herbal extracts on growth performance, gut microbiota and digestive function in newly weaned pigs. *Canadian Journal of Animal Science*.

[23] Oetting L. L., Utiyama C. E., Giani P. A., Ruiz U. D. S., Miyada V. S. (2006). Efeitos de extratos vegetais e antimicrobianos sobre a digestibilidade aparente, o desempenho, a morfometria dos órgãos e a histologia intestinal de leitões recém-desmamados. *Revista Brasileira de Zootecnia*.

[24] Zhou T. X., Zhang Z. F., Kim I. H. (2013). Effects of dietary *Coptis Chinensis* herb extract on growth performance, nutrient digestibility, blood characteristics and meat quality in growing-finishing pigs. *Asian-Australasian Journal of Animal Sciences*.

[25] El-Ashry M. A., El-Bordeny N. E., Khattab H. M., El-Sayed H. M. (2006). Effect of dietry supplemented with medicinal herbs on nutrient digestibility and some blood metabolites of buffalo calves. Egypt. *Journal of Nutritional Feed*.

[26] Qiao G. H., Zhou X. H., Li Y. (2012). Effect of several supplemental Chinese herbs additives on rumen fermentation, antioxidant function and nutrient digestibility in sheep. *Journal of Animal Physiology and Animal Nutrition*.

[27] Ghasemi S., Naserian A. A., Valizadeh R., Tahmasebi A. M., Vakili A. R., Behgar M. (2012). Effects of pistachio by-product in replacement of lucerne hay on microbial protein synthesis and fermentative parameters in the rumen of sheep. *Animal Production Science*.

[28] Weimer P. J., Waghorn G. C., Odt C. L., Mertens D. R. (1999). Effect of diet on populations of three species of ruminal cellulolytic bacteria in lactating dairy cows. *Journal of Dairy Science*.

[29] Mould F. L., Ørskov E. R. (1983). Manipulation of rumen fluid pH and its influence on cellulolysis in sacco, dry matter degradation and the rumen microflora of sheep offered either hay or concentrate. *Animal Feed Science and Technology*.

[30] Sung H. G., Kobayashi Y., Chang J., Ha A., Hwang I. H., Ha J. K. (2006). Low ruminal pH reduces dietary fiber digestion via reduced microbial attachment. *Asian-Australasian Journal of Animal Sciences*.

[31] Dalley D. E., Isherwood P., Sykes A. R., Robson A. B. (1997). Effect of *in vitro* manipulation of pH on magnesium solubility in ruminal and caecal digesta in sheep. *The Journal of Agricultural Science*.

[32] Wanapat M., Boonnop K., Promkot C., Cherdthong A. (2011). Effects of alternative protein sources on rumen microbes and productivity of dairy cows. *Maejo International Journal of Science and Technology*.

[33] Satter L. D., Roffler R. E. (1975). Nitrogen requirement and utilization in dairy cattle. *Journal of Dairy Science*.

[34] Soltan Y. A., Hashem N. M., Morsy A. S., El-Azrak K. M., El-Din A. N., Sallam S. M. (2018). Comparative effects of *Moringa oleifera* root bark and monensin supplementations on ruminal fermentation, nutrient digestibility and growth performance of growing lambs. *Animal Feed Science and Technology*.

[35] Liu C., Qu Y.-H., Guo P.-T., Xu C.-C., Ma Y., Luo H.-L. (2018). Effects of dietary supplementation with alfalfa (Medicago sativa L.) saponins on lamb growth performance, nutrient digestibility, and plasma parameters. *Animal Feed Science and Technology*.

[36] Jin Y. M., Jiang C., Zhang X. Q., Shi L. F., Wang M. Z. (2018). Effect of dietary *Urtica cannabina* on the growth performance, apparent digestibility, rumen fermentation and gastrointestinal morphology of growing lambs. *Animal Feed Science and Technology*.

[37] Zhou H., Wang C., Ye J., Chen H., Tao R. (2015). Effects of dietary supplementation of fermented *Ginkgo biloba* L. residues on growth performance, nutrient digestibility, serum biochemical parameters and immune function in weaned piglets. *Animal Science Journal*.

[38] Wang H., Chen Y., Liu H., Liu J., Makkar H. P., Becker K. (2011). Effects of replacing soybean meal by detoxified *Jatropha curcas* kernel meal in the diet of growing pigs on their growth, serum biochemical parameters and visceral organs. *Animal Feed Science and Technology*.

[39] Kim J. S., Ingale S. L., Lee S. H., Kim K. H., Lee J. H., Chae B. J. (2013). Effects of energy levels of diet and *β*-mannanase supplementation on growth performance, apparent total tract digestibility and blood metabolites in growing pigs. *Animal Feed Science and Technology*.

[40] Hosoda K., Kuramoto K., Eruden B., Nishida T., Shioya S. (2005). The effects of three herbs as feed supplements on blood metabolites, hormones, antioxidant activity, IgG concentration, and ruminal fermentation in Holstein steers. *Asian-Australasian Journal of Animal Sciences*.

[41] Hu C. H., Zuo A. Y., Wang D. G. (2011). Effects of broccoli stems and leaves meal on production performance and egg quality of laying hens. *Animal Feed Science and Technology*.

[42] Zhong R. Z., Fang Y., Zhou D. W., Sun X. Z., Zhou C. S., He Y. Q. (2018). Pelleted total mixed ration improves growth performance of fattening lambs. *Animal Feed Science and Technology*.

[43] Bauchart D. (1993). Lipid absorption and transport in ruminants. *Journal of Dairy Science*.

[44] Cornier M.-A., Dabelea D., Hernandez T. L. (2008). The metabolic syndrome. *Endocrine Reviews*.

[45] Kwon H. J., Kim Y.-Y., Choung S. Y. (2004). Effects of natural product extract on the fatty liver induced by alcohol diet in rats. *Journal of Health Science*.

[46] Li J., Yu L., Li N., Wang H. (2000). *Astragalus mongholicus* and *Angelica sinensis* compound alleviates nephrotic hyperlipidemia in rats. *Chinese Medical Journal*.

[47] Ming M., Guanhua L., Zhanhai Y., Guang C., Xuan Z. (2009). Effect of the *Lycium barbarum* polysaccharides administration on blood lipid metabolism and oxidative stress of mice fed high-fat diet *in vivo*. *Food Chemistry*.

[48] Kong X. F., Wu G. Y., Liao Y. P. (2007). Dietary supplementation with Chinese herbal ultra-fine powder enhances cellular and humoral immunity in early-weaned piglets. *Livestock Science*.

[49] Kallon S., Li X., Ji J. (2013). Astragalus polysaccharide enhances immunity and inhibits H9N2 avian influenza virus *in vitro* and *in vivo*. *Journal of Animal Science and Biotechnology*.

[50] Zhang X. H., Wang D. Y., Hu Y. L., Sun J. L. (2009). Immunologic enhancement of *Astragalus polysaccharide* (APS) on the humoral immunity of chicken. *Chinese Journal of Veterinary Science*.

[51] Guo L., Liu J., Hu Y. (2012). Astragalus polysaccharide and sulfated epimedium polysaccharide synergistically resist the immunosuppression. *Carbohydrate Polymers*.

[52] Che D., Adams S., Wei C., Qin G. X., Atiba E. M., Jiang H. (2018). Effects of *Astragalus membranaceus* fiber on growth performance, nutrient digestibility, microbial composition, VFA production, gut pH, and immunity of weaned pigs. *MicrobiologyOpen*.

